# Homologs of the LapD-LapG c-di-GMP Effector System Control Biofilm Formation by *Bordetella bronchiseptica*

**DOI:** 10.1371/journal.pone.0158752

**Published:** 2016-07-05

**Authors:** Nicolás Ambrosis, Chelsea D. Boyd, George A. O´Toole, Julieta Fernández, Federico Sisti

**Affiliations:** 1 Instituto de Biotecnología y Biología Molecular (IBBM)-CCT-CONICET-La Plata, Departamento de Ciencias Biológicas, Facultad de Ciencias Exactas, Universidad Nacional de La Plata, La Plata, Argentina; 2 Department of Microbiology and Immunology, Geisel School of Medicine at Dartmouth, Hanover, New Hampshire, United States of America; East Carolina University School of Medicine, UNITED STATES

## Abstract

Biofilm formation is important for infection by many pathogens. *Bordetella bronchiseptica* causes respiratory tract infections in mammals and forms biofilm structures in nasal epithelium of infected mice. We previously demonstrated that cyclic di-GMP is involved in biofilm formation in *B*. *bronchiseptica*. In the present work, based on their previously reported function in *Pseudomonas fluorescens*, we identified three genes in the *B*. *bronchiseptica* genome likely involved in c-di-GMP-dependent biofilm formation: *brtA*, *lapD* and *lapG*. Genetic analysis confirmed a role for BrtA, LapD and LapG in biofilm formation using microtiter plate assays, as well as scanning electron and fluorescent microscopy to analyze the phenotypes of mutants lacking these proteins. *In vitro* and *in vivo* studies showed that the protease LapG of *B*. *bronchiseptica* cleaves the N-terminal domain of BrtA, as well as the LapA protein of *P*. *fluorescens*, indicating functional conservation between these species. Furthermore, while BrtA and LapG appear to have little or no impact on colonization in a mouse model of infection, a *B*. *bronchiseptica* strain lacking the LapG protease has a significantly higher rate of inducing a severe disease outcome compared to the wild type. These findings support a role for c-di-GMP acting through BrtA/LapD/LapG to modulate biofilm formation, as well as impact pathogenesis, by *B*. *bronchiseptica*

## Introduction

*Bordetella bronchiseptica* is a Gram-negative bacterium that causes respiratory tract infections in mammals, atrophic rhinitis in pigs, kennel cough in dogs and snuffles in rabbits [1]. *B*. *bronchiseptica* has a variety of virulence factors that allow host infection. Each factor, such as pertactin, filamentous hemagglutinin, adenylate cyclase, the type three secretory system and lipopolysaccharide are likely to perform specific functions required for successful colonization [2–6].

The ability of *Bordetella spp*. to form biofilms has been reported in previous work, including the ability of *B*. *bronchiseptica* to form biofilms on abiotic surfaces regulated by the two-component system BvgAS [7,8]. As has been found for other biofilm-forming organisms, extracellular DNA (eDNA) and exopolysaccharide are important for biofilm formation by *B*. *bronchiseptica* [9,10]. Particularly in *Bordetella*, BvgAS-regulated factors, including the filamentous hemagglutinin and adenylate cyclase, may also participate in biofilm formation [7,11]. Static growth in intermediate nicotinic acid concentrations, a regulator of the BvgAS system, resulted in the best conditions for promoting biofilm formation [8]. Studies from our group and others indicate that biofilm is enhanced in the intermediate phase [7,12], however, there are conflicting findings regarding BvgAS-dependent biofilm regulation [8]. Furthermore, transcriptome analysis has shown that more than 33% of the *B*. *bronchiseptica* genes are differentially regulated during biofilm formation compared to planktonic culture [13]. Thus, further studies are needed to elucidate all factors affecting biofilm formation.

Sloan and colleagues observed biofilm-like structures *in vivo* in the nasal epithelium of *B*. *bronchiseptica* infected mice, and these communities expressed a polysaccharide essential for *in vivo* biofilm development [9]. The absence of polysaccharides, key factor for *in vitro* biofilm formation, also impaired infection suggesting that biofilm formation may also participate in host-pathogen interactions [9]. Thus, biofilm formation may play an important role in host-*B*. *bronchiseptica* interactions. Recently, we showed that bis-(3′-5′)-cyclic-dimeric guanosine monophosphate (c-di-GMP) regulates motility and biofilm formation in *B*. *bronchiseptica* [12]. C-di-GMP is a bacterial second messenger known to regulate a variety of cellular processes including biofilm formation, motility and virulence of bacterial pathogens [14,15]. Like in other bacteria where c-di-GMP-related functions have been studied, high c-di-GMP levels in *B*. *bronchiseptica* correlated with an enhanced biofilm formation phenotype [12]. However, in this previous study we did not uncover the mechanism by which c-di-GMP enhanced biofilm formation in *B*. *bronchiseptica*.

Here we confirm the role for the Bvg-regulated adhesin BrtA, a putative homolog of the well-characterized LapA adhesin of *Pseudomonas fluorescens*, in biofilm formation by *B*. *bronchiseptica*, as has recently been reported by Nishikawa and colleagues [16]. Furthermore, we show that the c-di-GMP receptor LapD and the LapD-regulated protease LapG also participate in biofilm formation by *B*. *bronchiseptica*, likely via the control of BrtA localization to the cell surface as has been reported for the LapA adhesin of *P*. *fluorescens*. We also present data that the appropriate control of BrtA via LapG appears to be important for modulating pathogenesis in a mouse model of infection. Thus, this work describes the basis of c-di-GMP-mediated control of biofilm formation in *B*. *bronchiseptica*, both *in vivo* and *in vitro*.

## Materials and Methods

### Ethics Statement

This study was performed in strict accordance with animal use protocols approved by Faculty of Sciences, National University of La Plata, Institutional Animal Care and Use Committee (IACUC), protocol number 007-00-15. All animals were properly anesthetized with isofluorane for bacterial inoculation, monitored daily and euthanized with isofluorane overdose if they met any early removal criteria (lethargy, hunched posture, or ruffled coat) to limit suffering.

### Microbiological Methods

For routine culture, *B*. *bronchiseptica* strains were grown on Bordet Gengou agar (BGA) (Difco) supplemented with 15% (vol/vol) defibrinated fresh sheep blood (BGA medium) at 36°C for 48 h, and replated on the same medium for 24 h. Liquid cultures were grown in Stainer-Scholte (SS) [17] medium at 36°C and 160 rpm. When appropriate BGA or SS was supplemented with kanamycin (80 μg ml^-1^), streptomyicin (200 μg ml^-1^) or gentamycin (50 μg ml^-1^). *P*. *fluorescens* and *E*. *coli* were grown in lysogeny broth (LB) [18] at 30°C and 37°C, respectively. When appropriate, antibiotics were added to the medium at the following concentrations: *E*. *coli*, 10 μg ml^-1^ gentamycin; *P*. *fluorescens*, 30 μg ml^-1^ gentamycin.

Replicative plasmids were introduced to *E*. *coli* and *B*. *bronchiseptica* by electroporation using standard techniques. Non-replicating plasmids were introduced into *B*. *bronchiseptica* by conjugation. The yeast strain InvSc1 (*Saccharomyces cerevisiae*; Invitrogen), was routinely cultured on YPD medium. When selecting for plasmids carrying the URA3 gene, yeast were grown on YNB with complete supplemental mixture minus uracil.

### Plasmid and Strain Construction

Strains and plasmids were constructed using standard molecular biology techniques and are listed in S1 Table. Oligonucleotides used in this study are listed in S2 Table. Detailed descriptions of strain and plasmid construction procedures can be found in the S1 Text.

### Biofilm Assays

Biofilm formation assays using static cultures were performed as described previously [12] from overnight cultures inoculated into SS liquid medium. Briefly, the culture was pipetted into glass tubes or wells of a sterile 96-well U bottom microtiter plate (polyvinylchloride, PVC) and incubated statically at 37°C. Nicotinic acid was added as indicated. After 24 hours, unless otherwise indicated, planktonic bacteria were removed and the attached cells were stained with 0.1% crystal violet (CV) solution. The stain was dissolved by adding 120 μl of 33% acetic acid solution. One hundred microliters of the dissolved stain solution was transferred to a new, flat-bottom microplate and then quantified by measuring OD at 595 nm.

### Scanning Electron Microscopy

Scanning electron microscopy pictures were obtained as described previously by Sisti *et al*. [12]. Briefly, *Bordetella* strains were cultured statically on glass coverslips partially submerged vertically in plastic tubes such that an air-liquid interface was established on the coverslip. After 24 h of incubation, the coverslip was removed and washed with sterile PBS, and the bacteria were fixed with 2.5% glutaraldehyde in PBS. Samples were dehydrated in a graded ethanol series (20, 50, 70, 90 and 100% for 60 min each), subjected to critical point drying using liquid carbon dioxide (EMITECH, K850) and sputter coated with gold (SPI Supplies). The surface topographies of the biofilm in A panels were visualized with a scanning electron microscope (ESEM FEI QUANTA 200), and detected with SDD (EDAX Apollo 40) camera. The surface topographies in B panels were visualized with a scanning electron microscope (Philips SEM 505), and the images were processed with the Image Soft Imaging System ADDA II.

### RNA Extraction for RT-PCR Studies

RNA extraction and RT-PCR studies were performed as described previously by our group [12]. RNA preparation from bacteria was performed using the Illustra RNAspin kit (GE, USA), and quantification of RNA was performed using a ND-1,000 NanoDrop spectrophotometer at 260 nm. Measurements of A260/280 were used to determine the purity of the RNA. The synthesis of cDNA was performed with a Reverse Transcription System kit (Promega) according to the manufacturer’s protocol using random primers. One microgram of RNA was used for each sample. The reaction was incubated at room temperature for 10 min, and reverse transcription was performed in a thermal cycler at 42°C for 15 min and 95°C for 5 min for a total of 35 cycles. PCR was performed with Kappa Taq from Biosystems. Samples that had not undergone the reverse transcription process were used as controls for the absence of genomic DNA in RT-PCR experiments. Primers employed are summarized in S2 Table, including the primers designed to amplify the intergenic region between the *lapA* and *lapG* genes. PCR analysis of internal fragment of *recA* gene, which is constitutively expressed, was used as control.

### LapA Localization

For Western and dot blot assays of the LapA protein, we used strains with internal HA-tag engineered into the sequence of the chromosomal *lapA* gene. Culture conditions, preparation of clarified cell extracts, and analysis of samples for LapA localization were performed as described previously without modification [19]. Detection of cell surface LapA by dot blotting was also performed as previously described [19,20].

### LapG Activity Assays

Bacterial cultures were grown overnight in LB, and clarified cell extracts were subsequently prepared by sonication (4×10 s on ice) in resuspension buffer, followed by centrifugation 12 min at 15,000 × *g*. As described previously [19], to assess cleavage of N-terminal domain of LapA (N-term-LapA), cell extracts *E*. *coli* carrying the pMini-LapA plasmid were mixed 1:1 with cell extracts from strains with and without a LapG-expressing plasmid, followed by incubation at room temperature for 30 min. When indicated, purified *P*. *fluorescens* LapG was added (750 ng of LapG; est. purity: 50%).

### Murine Respiratory Infection Model

Female BALB/c mice (originally from National Institute of Health (USA) and maintained at Faculty of Veterinary, National University of La Plata) 3 to 4 weeks of age were used as a model of *in vivo* respiratory infection by *B*. *bronchiseptic*a. Bacteria grown on BGA medium were resuspended and adjusted to approximately 10^7^ CFU ml^-1^ in PBS. Fifty microliters of bacterial suspension was delivered intranasally to each mouse via an air displacement pipette. At different times post-inoculation, three to four mice from each group were euthanized and their lungs and nose were removed aseptically. Tissues were homogenized in PBS, and appropriate dilutions were plated onto BGA medium to determine the number of viable bacteria present in the lungs and nose. Results presented in figures are from two independent experiments performed with at least four mice per strain per time point. Counts from euthanized mice were excluded from calculations, as described in more detail in the Results section.

### Statistical Analysis

All the results were compared by analysis of variance (ANOVA) followed by the Tukey test using Infostat software (Cordoba National University).

## Results

### Genes Coding for Lap-Like Proteins Are Present and Expressed in *B*. *bronchiseptica*

We searched the *B*. *bronchiseptica* genome for genes coding for proteins with properties similar to the LapA protein of *P*. *fluorescens*, including a type I secretion signal, a Von Willebrand factor type A domain (vWFA), an overall large size, a domain with sequence repeats, as well as *lapG-* and *lapD-*like genes found in close proximity to the candidate LapA-like protein(s). We performed a BLASTP search using ~1200 C-term amino acids of *P*. *fluorescens* LapA as the query against the available *B*. *bronchiseptica* RB50 genome. This portion of LapA includes the vWFA domain and type I secretion signal. The search identified a protein with ~30% identity to the query sequence; the identified protein contained a C-term domain of a putative hemolysin. Inspection of the complete amino acid sequence identified a protein with LapA-like properties in *B*. *bronchiseptica*—BB_RS05895.

**Fig 1:**
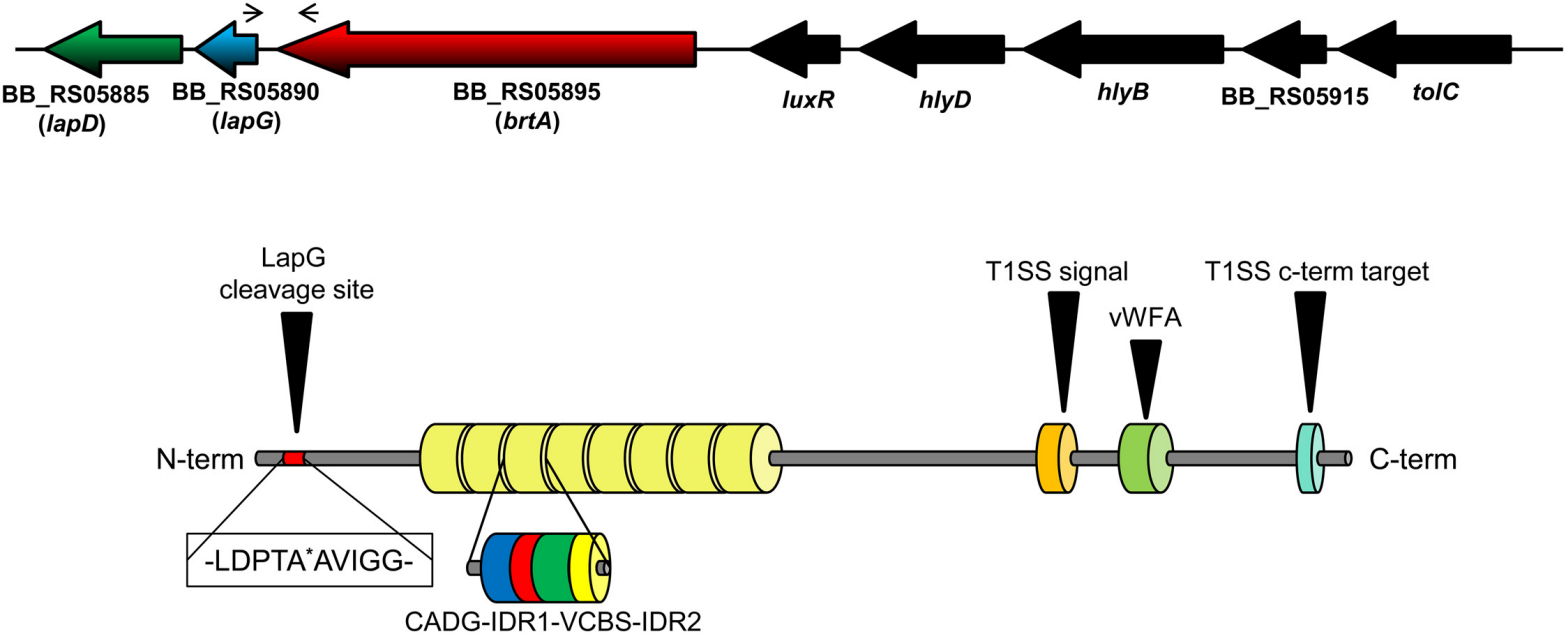
*B*. *bronchiseptica* LapA domain organization. Diagram showing the organization of the *lapD*, *lapG* and *brtA genes* in *B*. *bronchiseptica* (top) as well as the domain organization of the BrtA protein (bottom). The narrow arrows indicate the location of the primers used in the RT-PCR experiment. The thick, black arrow indicates the specific cleavage site for LapG. The type I secretion signal (T1SS) and T1SS target domains of BrtA are also shown (orange and cyan, respectively), as is the von Willebrand Factor A (vWFA) domain (light green). The repeat regions are indicated by the 8 yellow barrels. Domains found in repeated regions are also indicated: CADG domain (blue), VCBS domain (green) and IDR (interdomain region, red and yellow). The CADG and VCBS domain names are based on core conserved amino acid.

The BB_RS05895 ORF was recently described by Nishikawa and co-workers and named *brtA* [16], thus we use that nomenclature here. The *B*. *bronchiseptica* RB50 BrtA protein is predicted to be 3345 amino acids in length, with eight repeat regions. Each repeat region is ~200 amino acids long and is composed of distinct regions designated the CADG and VCBS (Dystroglycan-type cadherin-like domains and *Vibrio*, *Colwellia*, *Bradyrhizobium*, and *Shewanella* repeats superfamily respectively) domains based on conserved amino acid sequence motifs (Fig 1, S1 Fig). Interestingly, the number of repeats in BrtA vary among *B*. *bronchiseptica* strains. As described in S3 Table, the number of repeats varies between 2 and 15 with a median of 3 repeats. These differences in repeat length may impact host tropism, however no correlation was observed between isolate source and number of repeated regions (S3 Table). It is important to note that many draft genomes have incomplete BrtA sequences, likely due to high frequency of repeated sequences in this protein. Hence data regarding number of CADG-IDR1-VCBS-IDR2 repeats in BrtA homologs is not available for all genomes.

While BrtA architecture is similar to the LapA protein of *P*. *fluorescens* and the RtxA protein of *Legionella pneumophila* [21], the sequence of the repeat regions is distinct. Nishikawa and colleagues showed that the BrtA protein localizes to the surface of *B*. *bronchiseptica*, and using a *brtA* mutant strain, these workers also showed that BrtA is required for the adhesion to an abiotic (polystyrene) substratum but not to rat alveolar type 2 cells [16]. Upstream of BB_RS05895 we found BB_RS05890 and BB_RS05885, which show sequence similarity to the *P*. *fluorescens* LapG (48% identity) and LapD (30% identity) proteins, respectively. Based on their sequence as well as functional conservation to the *P*. *fluorescens* proteins (as described below), we decided to name BB_RS05890 and BB_RS05885 as *lapG* and *lapD*, respectively. We also identified a canonical predicted cleavage site for LapG (an Ala-Ala motif) in the N-terminal portion of BrtA (Fig 1) [21], indicating that BrtA cell surface localization is likely controlled by the LapG protease, a hypothesis we test further below.

**Fig 2:**
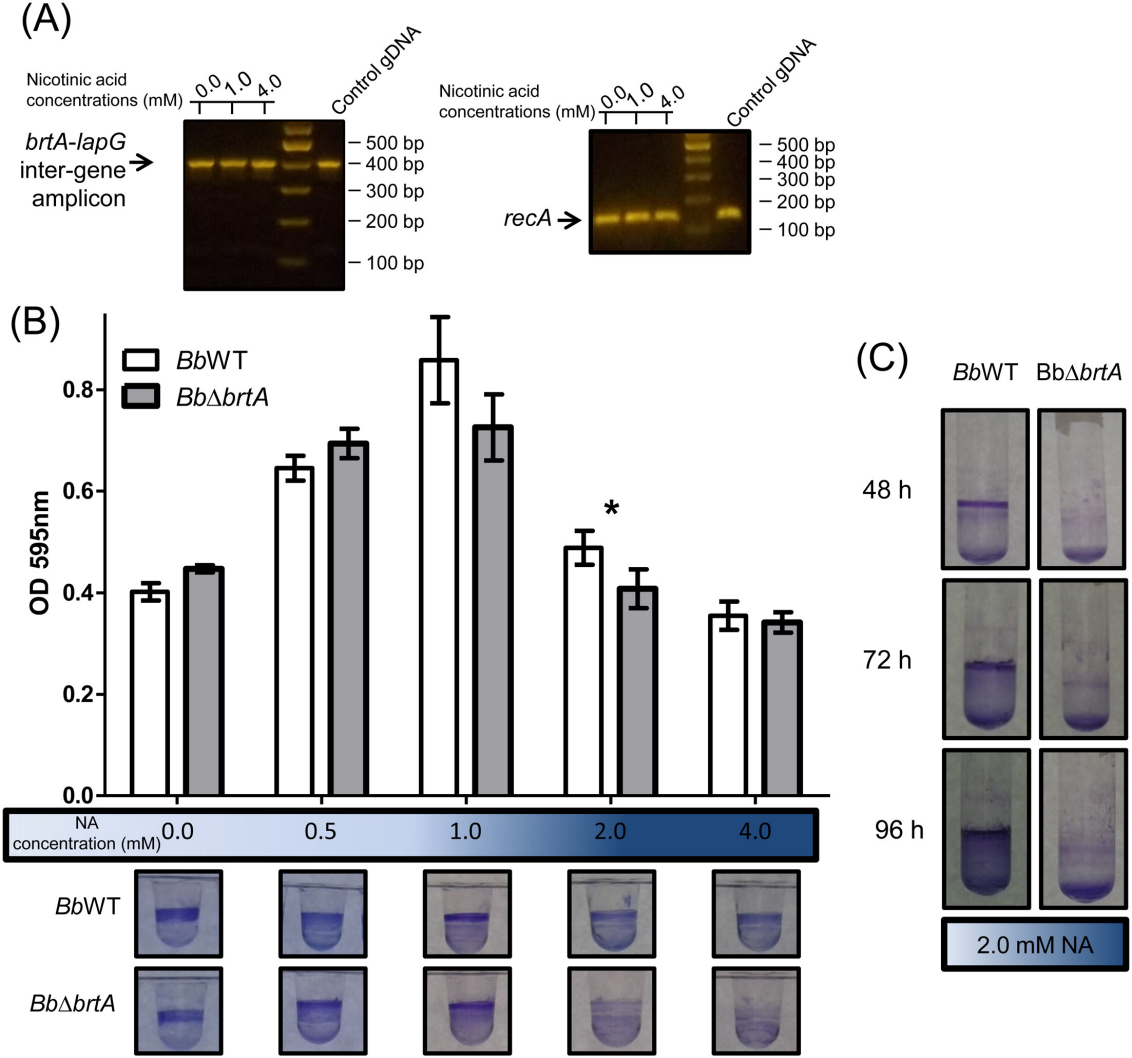
Loss of BrtA in *B*. *bronchiseptica* negatively impacts biofilm formation *in vitro*. (A) RT-PCR using *B*. *bronchiseptica* cDNA as template to amplify intergenic regions between the *lapA* and *lapG* genes. The *recA* amplicon was used as a positive control for the constitutively expressed *recA* gene. cDNA obtained from wild type *B*. *bronchiseptica* grown in different NA concentrations was employed in these assays. Note: the RT-PCR assays used are not quantitative, but are useful for detecting the presence/absence of a transcript. (B) Biofilm development by the *Bb*WT and *BbΔbrtA* strains cultured in SS medium either alone or supplemented with NA at the indicated concentration. Biofilm formation was assessed by PVC microtiter plate assays as described in the Materials and Methods. *, indicates a significant difference between the wild type and mutant with same concentration of NA, p<0.01. After incubation in static conditions, the medium was removed, the bacteria were stained with CV, and the extent of biofilm formation quantified. (C) Biofilm formation by the *Bb*WT and *BbΔbrtA* strains assessed in SS supplemented with NA 2.0 mM in glass tubes.

The gene organization in *B*. *bronchiseptica* suggested the *lapD*, *lapG and brtA* genes are part of an operon. Intergenic regions of 81 bp and 16 bp were found between the end of the *brtA* gene and start of the *lapG* gene and between the *lapG* and *lapD* genes, respectively. To test whether these genes are co-transcribed, we performed RT-PCR using primers that amplified the intergenic region between the *brtA* and *lapG* genes (Fig 1). Results presented in Fig 2A show that the *brtA* and *lapG* genes appear to be co-transcribed. Moreover, these genes appear to be transcribed in both virulent and avirulent phases, as indicated by the detection of the PCR product across a range of nicotinic acid (NA) concentrations.

We compared the amino acid sequence of the *B*. *bronchiseptica* LapD- and LapG-like proteins. LapD of *B*. *bronchiseptica* has cytosolic GGDEF and EAL domains that have been shown to be critical to the function of the LapD protein of *P*. *fluorescens* [19]. The *B*. *bronchiseptica* LapG-like protein is 48% identical to its *P*. *fluorescens* counterpart; sequence analysis shows that all the amino acids described by Chatterjee *et al*. as important for protease activity, as well as for binding to LapD and calcium [20] are conserved between the LapG proteins of *B*. *bronchiseptica* and *P*. *fluorescens*.

### BrtA Is Important for Biofilm Formation in a Surface-Dependent Manner

Recently BrtA was described as an avirulence factor involved in biofilm formation in *B*. *bronchiseptica* RB50 [16]. To confirm its role in biofilm formation, we deleted the *brtA* gene of *B*. *bronchiseptica* 9.73H^+^. The ability to form a biofilm was evaluated by the crystal violet (CV) biofilm assay using a 96-well polyvinylchloride (PVC) microtiter plates as described in the Material and Methods. We determined the amount of biofilm biomass formed as a function of NA concentration; this chemical serves as an environmental modulator of BvgAS activity [22]. As shown in Fig 2B, we observed a modest but significant reduction in the amount of biofilm formed by a mutant lacking the BrtA protein (*BbΔbrtA*) at 2 mM NA. In contrast, biofilm formation by the *Bb*Δ*brtA* strain was substantially reduced at 2 mM NA when biofilm formation was assessed on a hydrophilic surface (glass, Fig 2C). Taken together, our data indicate that BrtA differentially contributes to biofilm formation on different surfaces.

### *B*. *bronchiseptica* LapG Is a Protease that Cleaves the N-Terminus of BrtA

Newell and co-workers previously described the *P*. *fluorescens* mutant *PfΔlapG*, a strain with a clean deletion of the *lapG* gene and expressing a fully functional, HA-tagged variant of the LapA adhesin [20]. The lack of LapG in *P*. *fluorescens* results in a strain that forms a hyper-biofilm due to the enhanced levels of LapA on the surface of the cell [20].

**Fig 3:**
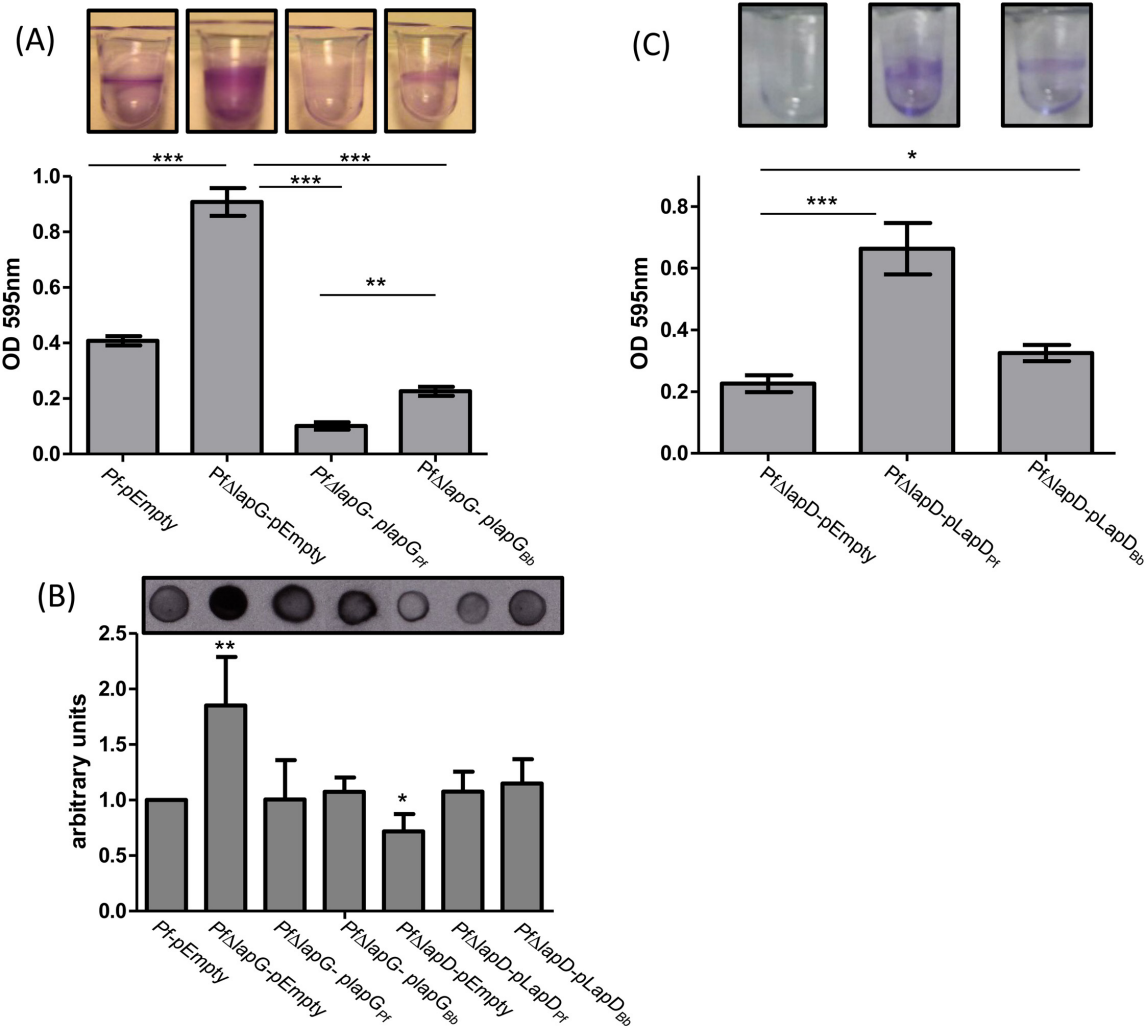
*B*. *bronchiseptica* LapG and LapD complement their respective *P*. *fluorescen*s mutations. (A) Biofilm formation by the *P*. *fluorescens ΔlapG* mutant (*PfΔlapG*) carrying plasmids expressing the *B*. *bronchiseptica* or *P*. *fluorescens lapG* genes was determined by the CV biofilm assay. (B) Cell surface levels of LapA as measured by dot blot. Shown is a representative dot blot assay (top) and quantification of the pixel density (n = 6 ± SD; bottom) to assess the level of cell surface LapA of the *P*. *fluorescens* strains expressing LapG or LapD, as well as indicated control strains. *, ** or *** indicate significant differences between strains, p<0.05, p<0.01 and p<0.005 respectively. (C) Biofilm formation by the *P*. *fluorescens ΔlapD* mutant (*PfΔlapD*) carrying plasmids expressing the *B*. *bronchiseptica* or *P*. *fluorescens lapD* genes was determined by the CV biofilm assay. *, ** or *** indicate significant differences between strains, p<0.05, p<0.01 and p<0.005 respectively.

We hypothesized that if the LapG-like protein of *B*. *bronchiseptica* shared function with LapG of *P*. *fluorescens*, expression of the *B*. *bronchiseptica* LapG protein would reduce the biofilm formed by the *PfΔlapG* mutant strain. The *B*. *bronchiseptica lapG*-like gene was cloned into a broad host vector, and this construct and the vector control were introduced into the wild-type *P*. *fluorescens* or the *PfΔlapG* mutant strain. Expression of the *B*. *bronchiseptica* LapG protein in the *PfΔlapG* strain significantly (p< 0.01) reduced the biofilm formed compared to the same mutant carrying pEmpty control vector (Fig 3A). Expression of the *P*. *fluorescens* LapG protein in the *PfΔlapG* mutant served as a positive control for this assay.

To confirm that the reduction in biofilm formation caused by the expression of the *B*. *bronchiseptica* LapG protein in the *PfΔlapG* mutant was due to a reduction in LapA on the cell surface, we performed the reported dot blot assay [19] to quantify cell-surface localized LapA protein in *P*. *fluorescens*. LapA was indeed reduced on the bacterial cell surface when *B*. *bronchiseptica* LapG was expressed in the *PfΔlapG* mutant (Fig 3B, first 4 bars), suggesting that LapG of *B*. *bronchiseptica* is indeed a protease that can target *P*. *fluorescens* LapA as a substrate.

**Fig 4:**
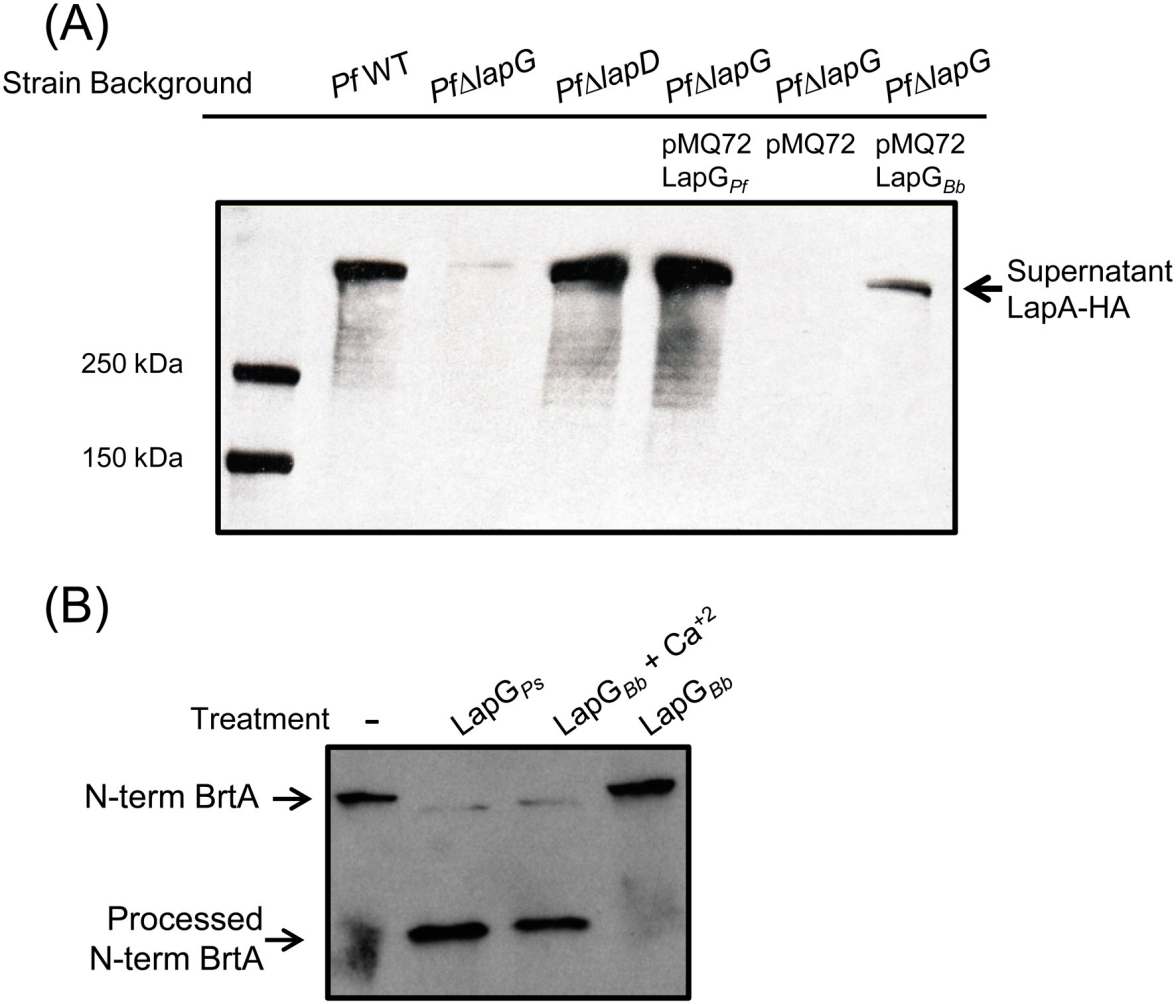
*B*. *bronchiseptica* LapG cleaves *P*. *fluorescen*s LapA and *B*. *bronchiseptica* BrtA. (A) Cleavage of LapA-HA from the surface of *P*. *fluorescens* cells with *B*. *bronchiseptica* LapG. Western blot with anti-HA antibodies were used to detect LapA-HA of *P*. *fluorescens* released into the supernatant fraction for each indicated strain. (B) Cleavage of N-terminal fragment of BrtA by *B*. *bronchiseptica* LapG *in vitro*. Samples correspond to reaction mixture of *E*. *coli* lysate expressing indicated protein or empty vector with *E*. *coli* lysate expressing the N-terminus of BrtA (N-term BrtA) incubated at room temperature for 1h. Western blot with anti-HA antibodies was used to detect N-term BrtA.

To confirm that *B*. *bronchiseptica* LapG is a protease that cleaves BrtA we performed biochemical assays using two different approaches. In the experiment shown in Fig 4A, the indicated *P*. *fluorescens* strain was transformed with a vector control (pMQ72), a plasmid carrying the LapG protease from *P*. *fluorescens* (LapG_*Pf*_) as a positive control, or *B*. *bronchiseptica* (LapG_*Bb*_). In these assays, we assess LapG activity by measuring the amount of LapA protein of *P*. *fluorescens* released into the supernatant; detecting supernatant LapA requires a functional LapG protease. The source of LapA in these studies is the chromosomally-encoded LapA modified with an HA-tag to facilitate its detection. As expected, in the wild type *P*. *fluorescens* strain (*Pf*WT, Fig 4A, lane 2), LapA protein can be detected in the supernatant but no LapA is detected in the *P*. *fluorescens* strain lacking LapG (*PfΔlapG*, Fig 4A, lane 3). As an additional control, we see abundant LapA in the supernatant in a *lapD* mutant strain (*PfΔlapD*, Fig 4A, lane 4); a *lapD* mutation results in high, unregulated LapG activity, and thus serves as an additional positive control for LapA release into the supernatant.

Our data indicate that *B*. *bronchiseptica* LapG can indeed substitute for the LapG protein of *P*. *fluorescens* as indicated by its ability to release LapA into the supernatant (Fig 4A, lane 7), albeit to a lesser extent than the *P*. *fluorescens* LapG protein (Fig 4A, lane 5). In the *P*. *fluorescens* strain lacking LapG (*PfΔlapG*) carrying the pMQ72 vector (lane 6), as expected, no LapA was detected in the supernatant. These data are consistent with the hypothesis that *B*. *bronchiseptica* LapG is a protease that can target cell-surface adhesins like LapA.

To extend the finding in Fig 4A to an *in vitro* system, an *E*. *coli* strain expressing *B*. *bronchiseptica* LapG was grown, concentrated and sonicated to generate a crude cell-free extract, as described in the Materials and Methods. Crude cell free extracts of *E*. *coli* containing the HA-tagged, N-term fragment of *B*. *bronchiseptica* BrtA (N-term BrtA, amino acids 1–330) were also generated. The crude extract containing *B*. *bronchiseptica* LapG was mixed in a 1:1 ratio with the extract containing the N-terminal fragment of *B*. *bronchiseptica* BrtA, incubated at room temperature for 1 hr, then analyzed by Western blotting using an anti-HA-tag antibody to detect processing of BrtA. As a positive control, LapG from *P*. *fluorescens* was shown to process N-term BrtA, and *B*. *bronchiseptica* LapG was also able to cleave the N-terminal fragment of *B*. *bronchiseptica* BrtA (Fig 4B). Interestingly, *B*. *bronchiseptica* LapG only cleaved BrtA in presence of 10 mM calcium. This finding is consistent with previous work showing that the LapG protein of *P*. *fluorescens* is a calcium-dependent enzyme [23]. Interestingly, Nishikawa and colleagues observed that treatment of *B*. *bronchiseptica* with EGTA, a calcium chelator, reduced biofilm formation; this reduction in biofilm formation may be due to the need for calcium to promote proper folding of some secreted proteins [24,25].

### *B*. *bronchiseptica* LapD Appears Partially Functional in *P*. *fluorescens*

The *B*. *bronchiseptica lapD*-like gene was cloned in a broad host vector to evaluate its ability to complement the phenotype of a *lapD* mutant of *P*. *fluorescens* (*PfΔlapD*). The *PfΔlapD* strain shows reduced biofilm formation and low LapA levels on the bacterial surface compared with the wild type [19]. When the *B*. *bronchiseptica lapD* gene was expressed in the *PfΔlapD* strain, partial complementation was observed in CV biofilm assay (Fig 3C). The level of cell surface LapA in the strain carrying the *B*. *bronchiseptica* LapD was increased to an extent similar to that observed when the *P*. *fluorescens lapD* gene was introduced into this strain (Fig 3B). We do not understand why the increase in cell surface LapA to wild-type levels in the strain carrying *B*. *bronchiseptica* LapD does not also result in an increase in biofilm formation to levels equivalent to the wild-type strain.

### The Deletion or Over-Expression of LapG Alters Biofilm Formation by *B*. *bronchiseptica*

Previous studies of *P*. *fluorescens* showed that unregulated LapG expression results in increased cleavage of LapA and the release of this adhesin into the supernatant, results in reduced biofilm formation. In contrast, deleting the *lapG* gene of *P*. *fluorescens* results in enhanced levels of LapA on the cell surface and increased biofilm formation [20]. Thus, we assessed the effect of expressing LapG from a plasmid and deleting the gene from the chromosome on biofilm formation by *B*. *bronchiseptica* as a function of NA, an *in vitro* regulator of biofilm formation.

**Fig 5:**
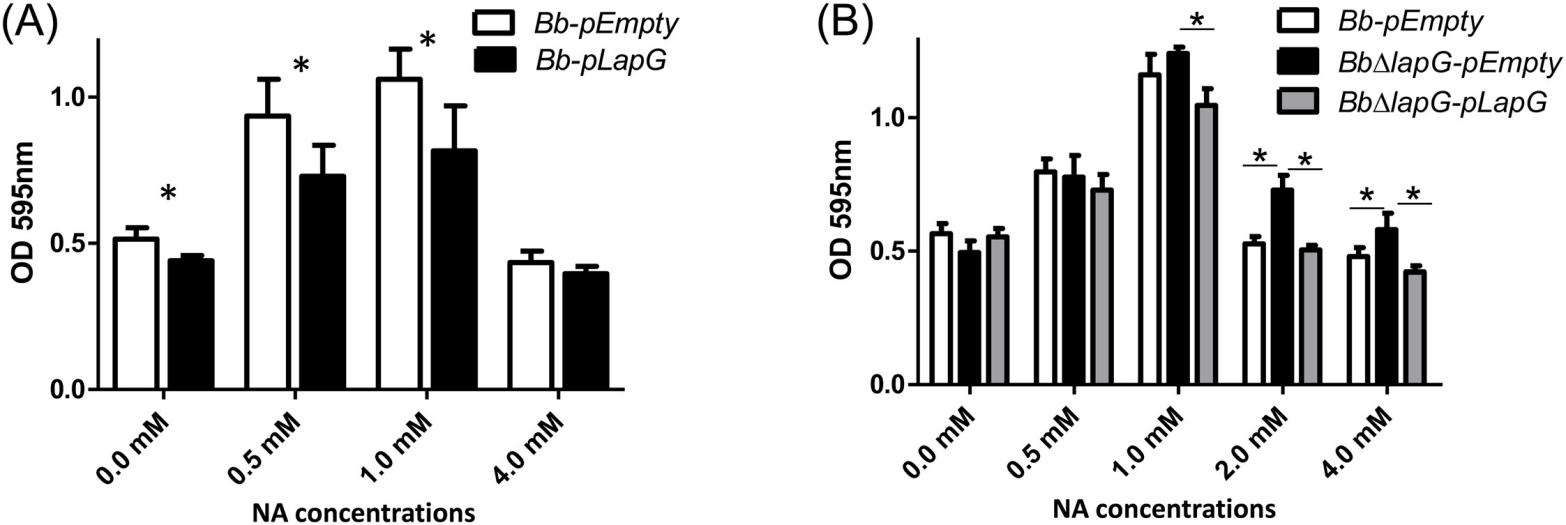
LapG regulates biofilm formation by *B*. *bronchiseptica*. (A) Biofilm formation in different NA concentrations by the wild type carrying a vector control (*Bb*-pEmpty) or a plasmid over-expressing *B*. *bronchiseptica* LapG (*Bb-lapG*). *, indicates a significant difference versus the *Bb*-pEmpty in same NA concentration, p<0.001. (B) Biofilm formation by the wild-type strain carrying a vector control (*Bb*-pEmpty), a strain with a deletion of the *lapG* gene carrying a vector control (*BbΔlapG*-pEmpty), and the *BbΔlapG* mutant complemented with a wild-type copy of the *B*. *bronchiseptica lapG* gene (*BbΔlapG*-pLapG). Biofilm formation was assessed using the microtiter plate assays as described in the Materials and Methods. *, indicates a significant difference in the indicated comparisons, p<0.001.

We hypothesized that expression of *B*. *bronchiseptica* LapG under a constitutive promoter would decrease biofilm formation in *B*. *bronchiseptica*. As expected, LapG overexpression resulted in significantly (p<0.001) reduced biofilm formation (Fig 5A) but only at low concentrations of NA added 0, 0.5 and 1 mM. At the highest concentration of NA tested (4.0 mM), where the extent of biofilm formation was the lowest, expression of the *B*. *bronchiseptica* LapG did not further reduce biofilm formation.

Based on the work in *P*. *fluorescens*, we would expect a *B*. *bronchiseptica* strain with a deletion of the *lapG* gene to develop very robust biofilms compared to the wild type. As expected, the *B*. *bronchiseptica* strain lacking the *lapG* gene (*BbΔlapG*) showed more biofilm formation compared to wild type at the higher concentrations of NA tested, and this defect could be complemented *in trans* by a wild-type copy of LapG_*Bb*_ (Fig 5B). This phenotype was observed on either a PVC (Fig 5B) or glass surface (not shown).

**Fig 6:**
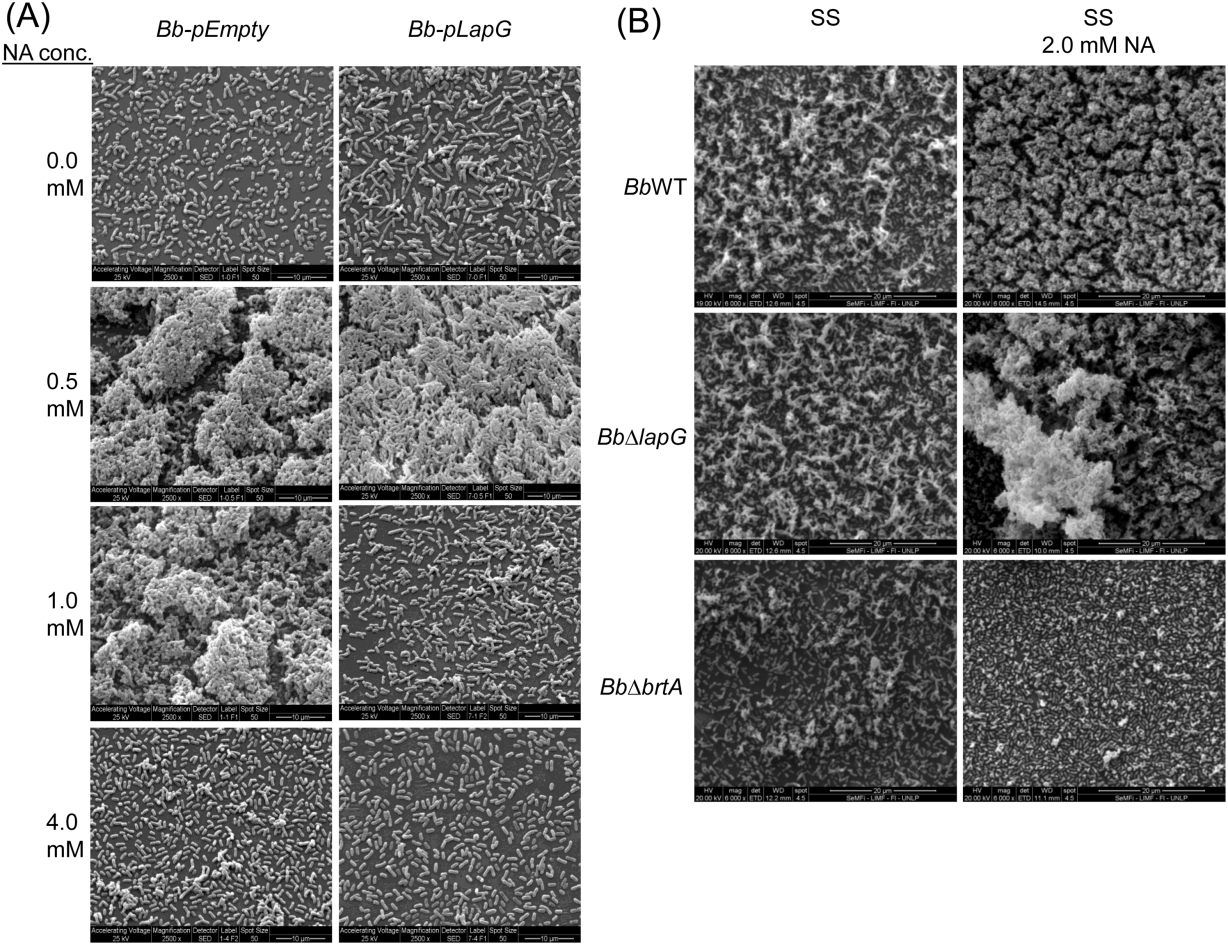
SEM images of *B*. *bronchiseptica* biofilms. (A) The strains *Bb*-pEmpty (left column of panels) or *Bb*-pLapG (right column of panels) were grown on vertically submerged coverslips in SS medium alone or supplemented with indicated NA concentrations. After 24 h of growth, biofilms formed at the air–liquid interface were visualized by SEM. (B) Indicated strains were grown on vertically submerged coverslips in SS medium alone (left column of panels) or SS supplemented with 2.0 mM NA (right column of panels).

The observations in Fig 5 using the microtiter plate assay were confirmed by SEM analysis of biofilm structures formed in the air-liquid interface using glass as an abiotic surface. Fig 6 shows representative SEM images of the biofilms formed by strains over-expressing LapG compared to a vector control (Fig 6A) and in a strain lacking the *lapG* gene compared to the wild type (Fig 6B). As a control, deletion of the *brtA* gene reduced (but did not eliminate) the biofilm formed under these conditions (Fig 6B). Similar results were observed when fluorescence microscopy was employed (S2 Fig).

### LapG May Contribute to Infection *In Vivo*

**Fig 7:**
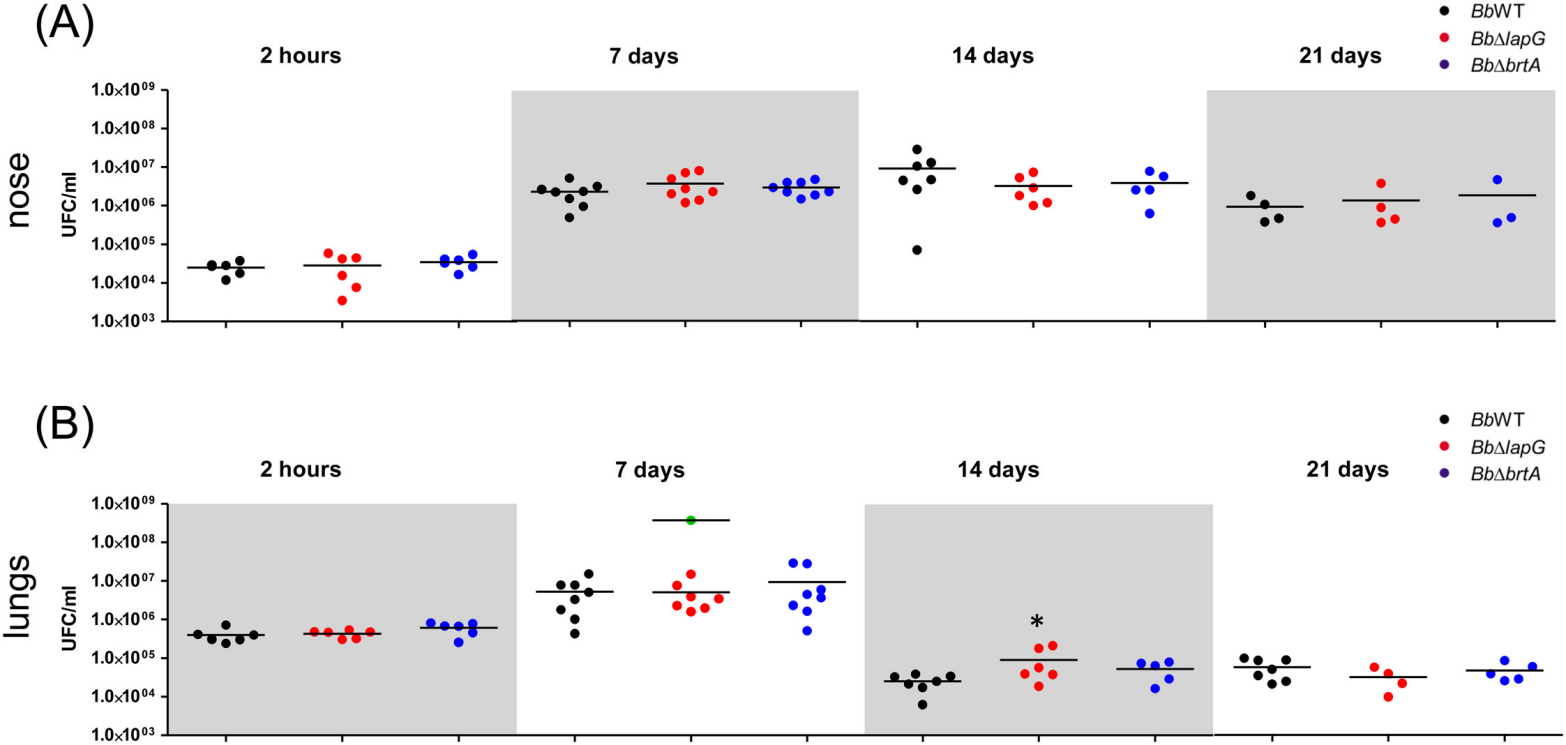
*B*. *bronchiseptica* infection in a mouse model. Comparison of *B*. *bronchiseptica* bacterial burden in the mouse nose (A) and lungs (B) were determined as described in the Materials and Methods after the indicated days post-infection. Bacteria were intranasally inoculated into external nares with an air displacement pipette (5 x 10^5^ CFU in 50 μl). Green dots correspond to CFU from euthanized mice infected with *BbΔlapG*. * indicates a significant difference compared to the CFU determined in mice infected with wild type strain, p<0.01.

Biofilm formation on epithelial cells in the nose is thought to be a key early step in *Bordetella* infection [9]. Given the role of BrtA and LapG for *in vitro* biofilm formation, we hypothesized that these proteins might also play a role during colonization *in vivo*. We intranasally inoculated suspensions of the wild type or mutant *B*. *bronchiseptica* strains into BALB/c mice, and the infection progress was evaluated for 21 days in the nose and lungs (Fig 7).

Mice were infected with wild type *B*. *bronchiseptica* as previously described [4]. Wild type bacteria persisted in lung and nose mice after 21 days post infection. A typical *B*. *bronchiseptica* infection was observed; lung burden increased during the first 7 days and this microbe was gradually reduced across the remainder of the experiment. Our analysis indicates that BrtA does not contribute to bacterial burden at any stage during infection in either body site evaluated (Fig 7). For the strain lacking the *lapG* gene (*BbΔlapG*), there was a small but statistically significant increase in bacterial burden at 14 days for those mice that survived until the end of the experiment, but no significant difference was detected at any other time point (Fig 7).

Interestingly, across all experiments, 25% of *BbΔlapG* infected mice presented symptoms of illness (indicated by ruffled fur, labored breathing, and diminished responsiveness) and were euthanized to avoid suffering (S3 Fig). Lungs of these euthanized mice carrying the *BbΔlapG* strain had on average 3 x 10^8^ CFU ml^-1^, a significant increase of 1–2 log over the wild type (7 x 10^6^ CFU ml^-1^) and the mice carrying the *BbΔlapG* mutant that survived until the end of the experiment. These data indicated the possibility that loss of LapG function by *B*. *bronchiseptica* could contribute to enhanced virulence, at least in some circumstances.

## Discussion

In this work we analyze the BrtA protein of *B*. *bronchiseptica*, a predicted adhesin based on its similarity in domain structure to the LapA adhesin of *P*. *fluorescens*. We confirm the recent finding of Nishikawa and colleagues [16] that BrtA participates in biofilm formation by *B*. *bronchiseptica*, and build on this recent study by identifying and characterizing homologs of the LapD and LapG proteins of *P*. *fluorescens*. In *P*. *fluorescens*, LapD and LapG participate in the regulation of cell surface localization of the LapA protein; we show that the *B*. *bronchiseptica* LapD and LapG proteins likely play a similar role for BrtA. We also demonstrate that loss of BrtA results in a mild biofilm defect on plastic, and a much more severe defect in biofilm formation on glass, a hydrophilic substrate. BrtA, while having limited sequence similarity to the LapA protein of *P*. *fluorescens* shares a number of conserved features, including T1SS and vWFA domains, and the conserved Ala-Ala motif near its N-terminus that serves as the site of proteolysis in LapA for the LapG protein of *P*. *fluorescens*.

In *P*. *fluorescens*, the localization of the LapA protein to the surface of the cell is postranscriptionally regulated by LapG. LapG is a periplasmic protease that targets the N-terminus of LapA and cleaves this adhesin at a conserved Ala-Ala motif, resulting in release of LapA into the supernatant. Loss of LapA results in a reduction in biofilm formation by *P*. *fluorescens*. The LapD protein of *P*. *fluorescens*, in response to c-di-GMP levels in the cytoplasm, regulates LapG’s ability to target LapA by sequestering LapG via a protein-protein interaction mechanism [20,26]. The studies presented here indicate that the LapD and LapG proteins of *B*. *bronchiseptica* likely play a similar role in the context of BrtA. Our *in vivo* and *in vitro* studies, including complementation assays, support the conclusion that LapG is a protease that targets the N-terminal domain of BrtA for cleavage, thereby negatively impacting biofilm formation. Similarly, our studies implicate LapD in the control of LapG in *B*. *bronchiseptica*.

An interesting observation from our SEM and fluorescence microscopy studies was that in a mutant lacking BrtA, biofilm formation was reduced but not eliminated. This observation suggested that there may be at least one other adhesin contributing to biofilm formation on abiotic surfaces. Previous reports implicated FHA and the adenylate cyclase enzyme as involved in biofilm formation by *Bordetella* [7,11], further work should be directed at assessing the role of these proteins (or others) in the context of a strain lacking BrtA. Interestingly, BrtA appeared to play no detectable role in disease progression in a mouse model of infection; there was no change in bacterial burden in the nose or lungs of mice for up to 21 days post-infection in the *brtA* mutant strain compared to the wild type. This finding is consistent with the work of Nishikawa and colleagues who showed that a *brtA* mutant of *B*. *bronchiseptica* showed no defect in a model of rat nasal septum and tracheal colonization. Given that BrtA plays a role in biofilm formation on abiotic surfaces, we propose that a major role for this adhesion might be in environmental persistence as the microbe transits between hosts.

A strain of *B*. *bronchiseptica* lacking LapG function, which presumably has increased cell-surface BrtA based on our *in vitro* studies, when infecting mice, yielded two distinct phenotypes. In 75% of the animals, infection was maintained for 21 days with a bacterial burden similar to the wild type, with the exception of a small but significant increase in bacterial numbers in lungs at day 14 post-infection. However, 25% of the mice infected with the *B*. *bronchiseptica lapG* mutant showed increased disease symptoms that resulted in the need to euthanize these animals typically between days 4–9. These data may indicate that BrtA, when present in larger levels on the cell surface can enhance virulence, at least in some animals. However, given the lack of impact upon loss of BrtA function in this infection model, it is also possible that LapG may have (at least) one other target in *B*. *bronchiseptica*. Distinguishing between these possibilities should be explored in future studies.

Overall, we have established that the LapD/LapG system of this pathogen likely functions in an analogous manner to the well-described system in *P*. *fluorescens*. In *B*. *bronchiseptica*, the target of the LapD/LapG system appears to be BrtA, a LapA-like protein that has a clear role in biofilm formation on abiotic surfaces. The role of the LapD/LapG system in a mouse model of infection is less clear—additional investigation will be required to dissect the role (if any) of BrtA and the LapD/LapG system in host-pathogen interactions.

## Supporting Information

S1 Fig Weblogo diagram of the repeated regions (CADG-IDR1-VCBS-IDR2) in BrtA. A consensus sequence among the eight repeat regions in BrtA is shown.
(TIF)

S2 Fig Fluorescent microscopy of *B*. *bronchiseptica* biofilm on glass surface. *Bordetella* strains were cultured on glass coverslips as described for the SEM studies. After 12 hr of incubation the coverslips were washed with sterile PBS, and the bacteria were fixed with 4% formaldehyde in PBS. The samples were observed in a Nikon Ti-U Fluorescence Microscope, and the images were obtained with a Nikon Digital Sight DS Ri1 camera using the Nis-Elements software. White bars indicate 100 μm.
(TIF)

S3 Fig Survival of mice infected with wild type or *lapG* mutant *B*. *bronchiseptica*. Kaplan Meier survival curve is represented for mice infected with *Bb*WT (blue line) or *BbΔlapG* (red line). The difference between the survival curves was analyzed by the log-rank test (z = 1.98; p = 0.047 with 95% confidence).
(PDF)

S1 Table Strains and plasmids used in this study. (DOCX)

S2 Table Oligonucleotides used in this study. (DOCX)

S3 Table Number of repeats domains in sequenced *B*. *bronchiseptica* genomes. (PDF)

S1 Text Detailed Materials and Methods. (DOCX)
