# Supplementary figures and images for: Homologs of the LapD-LapG c-di-GMP Effector System Control Biofilm Formation by Bordetella bronchiseptica
Source: PLoS One. 2016 Jul 5;11(7):e0158752. doi: 10.1371/journal.pone.0158752 (PMC4933386; doi:10.1371/journal.pone.0158752)

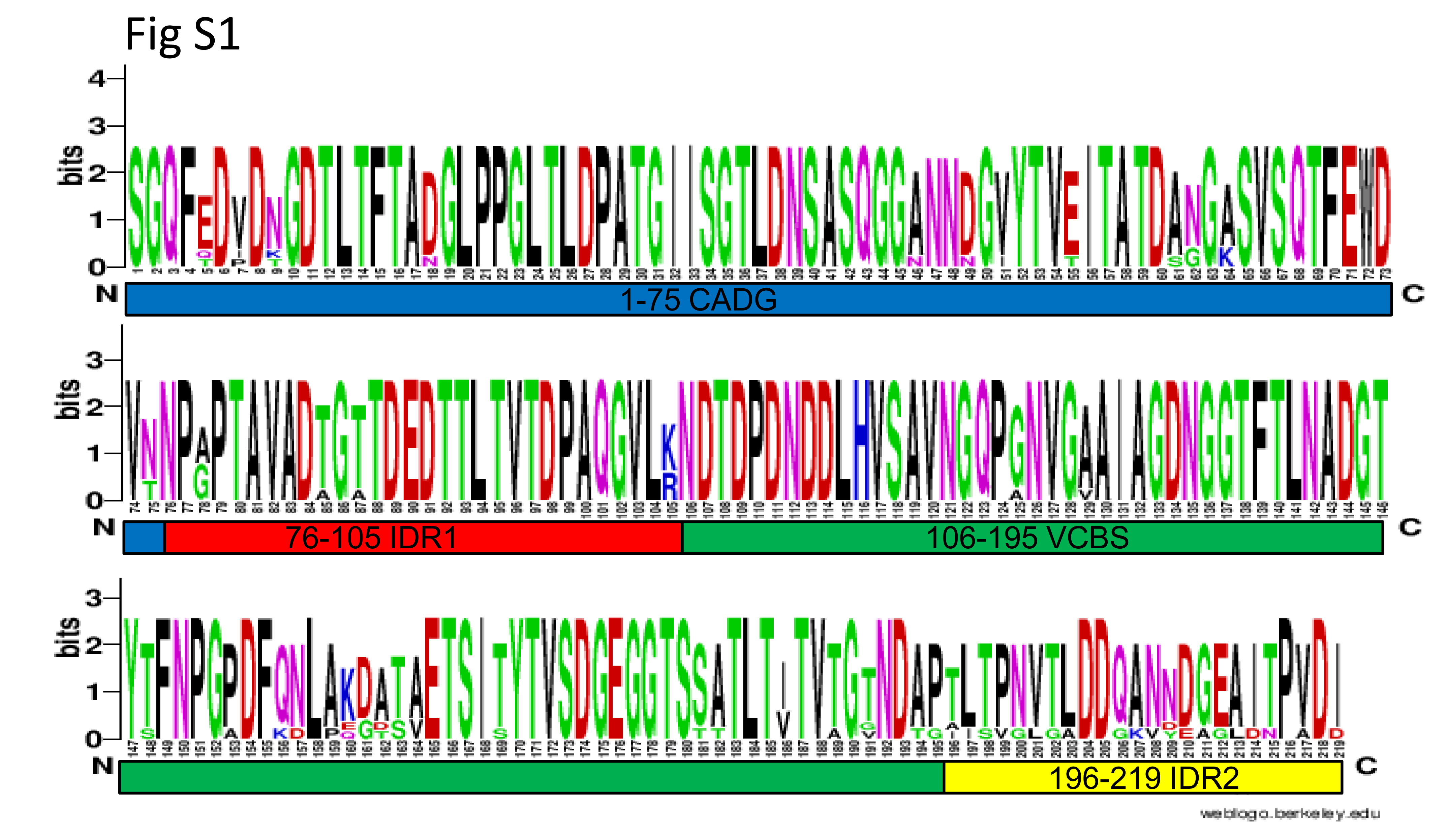

Supplement: S1 Fig — A consensus sequence among the eight repeat regions in BrtA is shown. (TIF) [file pone.0158752.s001.tif]

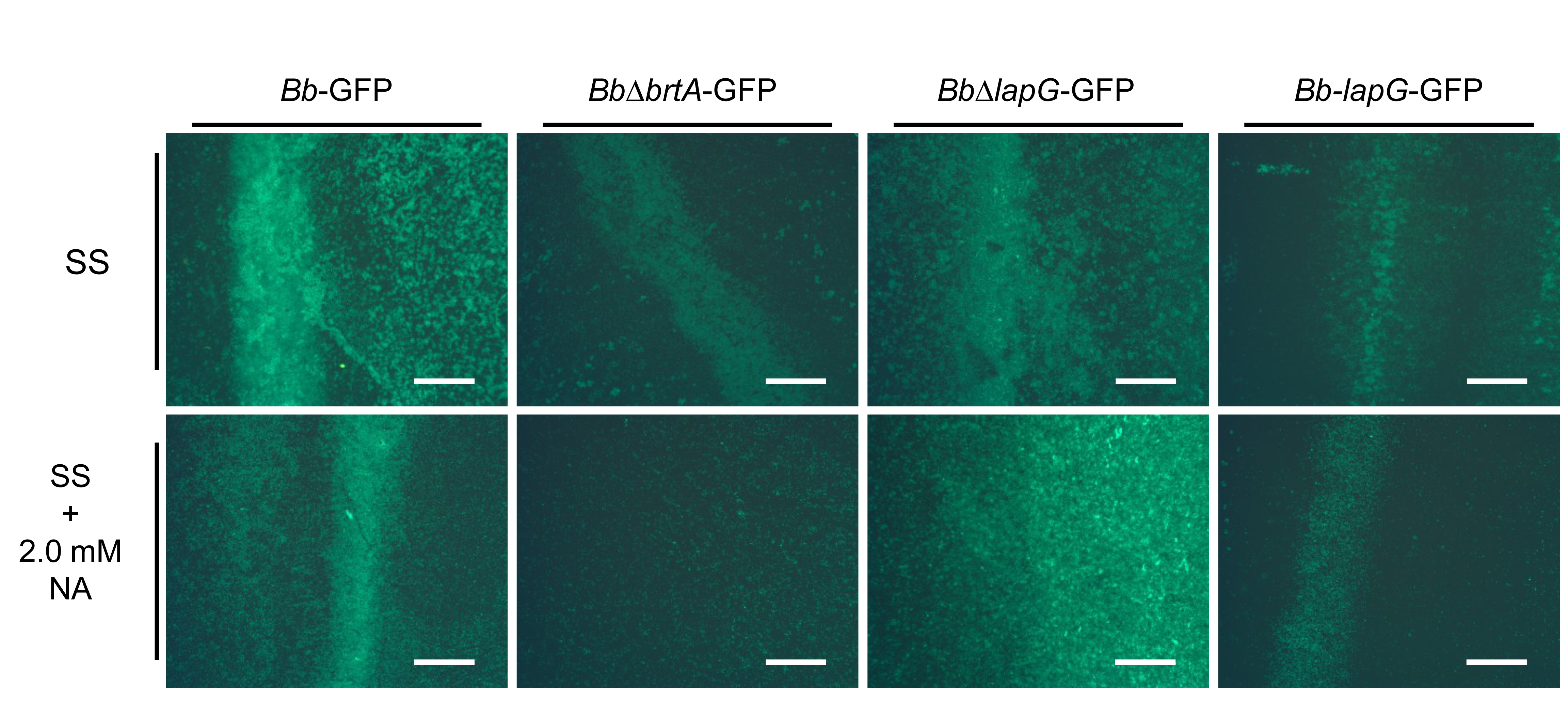

Supplement: S2 Fig — Bordetella strains were cultured on glass coverslips as described for the SEM studies. After 12 hr of incubation the coverslips were washed with sterile PBS, and the bacteria were fixed with 4% formaldehyde in PBS. The samples were observed in a Nikon Ti-U Fluorescence Microscope, and the images were obtained with a Nikon Digital Sight DS Ri1 camera using the Nis-Elements software. White bars indicate 100 μm. (TIF) [file pone.0158752.s002.tif]

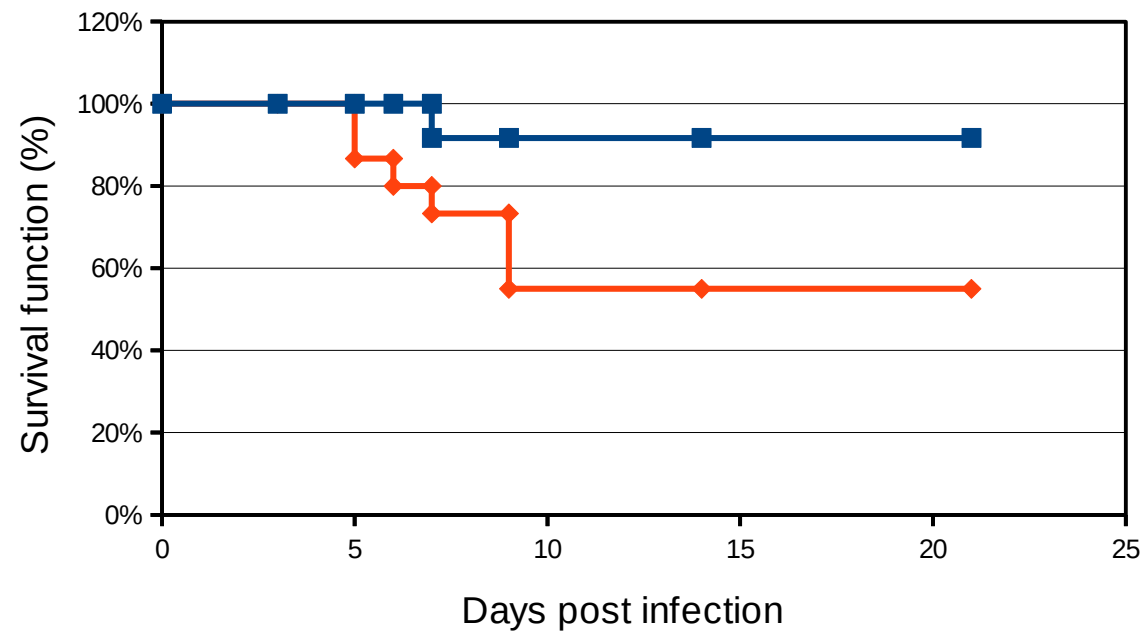

Supplement: S3 Fig — Kaplan Meier survival curve is represented for mice infected with BbWT (blue line) or BbΔlapG (red line). The difference between the survival curves was analyzed by the log-rank test (z = 1.98; p = 0.047 with 95% confidence). (PDF) [file pone.0158752.s003.pdf]
