# Supplementary material for: Homologs of the LapD-LapG c-di-GMP Effector System Control Biofilm Formation by Bordetella bronchiseptica
Source: PLoS One. 2016 Jul 5;11(7):e0158752. doi: 10.1371/journal.pone.0158752 (PMC4933386; doi:10.1371/journal.pone.0158752)
Supplement: S1 Table — (DOCX) [file pone.0158752.s004.docx]

**TABLE S1. Plasmids and strains used in this work.**

|  | **Description** | **Source or reference** |
| --- | --- | --- |
| Plasmids | | |
| pK18*mob*SacB | allelic replacement; *sacB* KmR | [9] |
| pK18SacBF1F2LapG | allelic replacement construct for Δl*apG* mutant. | This work |
| pK18SacBF1F2*brtA* | allelic replacement construct for Δ*brtA* mutant. | This work |
| pBBR1MCS-5 | Broad range vector. GmR | [14] |
| pBBR1MCS-5-*npt*II | pBBR1MCS-5 with *npt*II promotor | [8] |
| pBBR1MCS-5-*npt*BB1185 | pBBR1MCS-5-*npt*II with BB1185downstream promotor | This work |
| pMQ30 | allelic replacement; *sacB aacC1* ColE1 *oriT* CEN4 URA3 | [1] |
| pMQ30F1F2*lapG* | pMQ30 containing the *lapG* upstream and downstream region. | This work |
| pMQ30F1F2*brtA* | pMQ30 containing the *brtA* upstream and downstream region. | This work |
| pMQ72 | *Pseudomonas* expression vector, GmR | [1] |
| pMQ72*lapG*Bb | pMQ72 containing *lapG* form *B. bronchiseptica.* | This work |
| pMQ72*lapD*Bb | pMQ72 containing *lapD* form *B. bronchiseptica.* | This work |
| pNTerm-*brtA* *B. bronchiseptica* | pMQ72 expressing Nterm-*brtA*-HA from *B. bronchiseptica* | This work |
| pGFLIP | *Tn*7-based vector with PS12-*gfp* and *npt*II flanked by FRT sequences and *flp* 3’ to the MCS; Apr, Kmr (conditional) | [3] |
| pTNS3 | *Tn*7 transposase expression vector containing *tnsABCD*; Apr | [4] |
|  | | |
| *Bordetella bronchiseptica* strains | | |
| BbWT | Wild type strain. 9.73H+. SmR | [5] |
| RB50 | Wild type strain. SmR | [6] |
| RBX9F | RB50 with an unmarked deletion of *fhaB* and *fimA-fhaB* intergenic region | [7] |
| *Bb*-pEmpty | *Bb*9.73H+ wild type strain with pBBR1MCS-5-*npt*II | [8] |
| RB50-pEmpty | RB50 wild type strain with pBBR1MCS-5-*npt*II | This work |
| *Bb*-plapG | *Bb*WT expressing *lapG* | This work |
| RB50-plapG | RB50 expressing *lapG* | This work |
| RBX9F-plapG | RBX9 expressing *lapG* | This work |
| *Bb*ΔlapG | *Bb*WT with an unmarked deletion of *lapG* | This work |
| *Bb*ΔbrtA | *Bb*WT with an unmarked deletion of *brtA* | This work |
| *Bb*-GFP | *Bb*WT with PS12-*gfp* integrated at *attTn*7 | This work |
| *Bb*ΔlapG-GFP | *Bb*Δ*lapG* with PS12-*gfp* integrated at *attTn*7 | This work |
| *Bb*ΔbrtA-GFP | *Bb*Δ*brtA* with PS12-*gfp* integrated at *attTn*7 | This work |
| *Bb*-lapG-GFP | *Bb*-*lapG* with PS12-*gfp* integrated at *attTn*7 | This work |
|  | | |
| *Pseudomonas fluorescens* strains | | |
| *Pf*-*lapA*-HA | *P. fluorescens* Pf0-1 expressing *brtA*-HA, inserted after residue 4093 | [10] |
| *Pf*Δ*lapG* | *P. fluorescens* Pf0-1 with an unmarked deletion of *lapG* | [2] |
| *Pf*Δ*lapD* | *P. fluorescens* Pf0-1 with an unmarked deletion of *lapD* | [11] |
| *Pf*-pEmpty | *P. fluorescens* Pf0-1 with pMQ72 | This work |
| *Pf*Δ*lapD*-pEmpty | *Pf*Δ*lapD* with pMQ72 | This work |
| *Pf*Δ*lapG*-pEmpty | *Pf*Δ*lapG* with pMQ72 | This work |
| *Pf*Δ*lapD*-p*lapD*Pf | *Pf*Δ*lapD* expressing *lapD* from *P. fluorescens* | [11] |
| *Pf*Δ*lapD*-p*lapG*Pf | *Pf*Δ*lapG* expressing *lapG* from *P. fluorescens* | [11] |
| *PfΔlapD*-p*lapD*Bb | *Pf*Δ*lapD* expressing *lapD* from *B. bronchiseptica* | This work |
| *Pf*Δ*lapD*-p*lapG*Bb | *Pf*Δ*lapG* expressing *lapG* from *B. bronchiseptica* | This work |
|  | | |
| *Escherichia coli* strains | | |
| DH5a | *supE44 lacU169*(80*lacZ*M15) *hsdR17 thi-1 relA1 recA1* | [12] |
| S17-1 | (λ*pir*); *thi pro hsdR hsdM*+ Δ*recA* RP4-2::Tc*Mu*-Km::*Tn*7 | [15] |
| RH03 | Conjugation strain; Kms, DAP auxotroph | [13] |

**Literature Cited**.

1. Shanks RMQ, Caiazza NC, Hinsa SM, Toutain CM, O’Toole GA. *Saccharomyces cerevisiae*-based molecular tool kit for manipulation of genes from gram-negative bacteria. Appl Environ Microbiol. 2006;72: 5027–36. doi:10.1128/AEM.00682-06

2. Newell PD, Boyd CD, Sondermann H, O’Toole GA. A c-di-GMP effector system controls cell adhesion by inside-out signaling and surface protein cleavage. PLoS Biol. 2011;9: e1000587. doi:10.1371/journal.pbio.1000587

3. Byrd MS, Mason E, Henderson MW, Scheller E V, Cotter PA. An improved recombination-based in vivo expression technology-like reporter system reveals differential *cyaA* gene activation in *Bordetella species*. Infect Immun. 2013;81: 1295–1305. doi:10.1128/IAI.01445-12

4. Choi K-H, Mima T, Casart Y, Rholl D, Kumar A, Beacham IR, et al. Genetic tools for select-agent-compliant manipulation of *Burkholderia pseudomallei*. Appl Environ Microbiol. 2008;74: 1064–75. doi:10.1128/AEM.02430-07

5. Gueirard P, Guiso N. Virulence of *Bordetella bronchiseptica*: role of adenylate cyclase-hemolysin. Infect Immun. 1993;61: 4072–4078.

6. Cotter P a, Miller JF. BvgAS-mediated signal transduction: analysis of phase-locked regulatory mutants of *Bordetella bronchiseptica* in a rabbit model. Infect Immun. 1994;62: 3381–90.

7. Mason E, Henderson MW, Scheller E V, Byrd MS, Cotter PA. Evidence for phenotypic bistability resulting from transcriptional interference of *bvgAS* in *Bordetella bronchiseptica*. Mol Microbiol. 2013;90: 716–733. doi:10.1111/mmi.12394

8. Sisti F, Ha D-G, O’Toole GA, Hozbor DF, Fernandez J. Cyclic-di-GMP signaling regulates motility and biofilm formation in *Bordetella bronchiseptica*. Microbiology. 2013;159: 869–879. doi:10.1099/mic.0.064345-0

9. Schäfer A, Tauch A, Jäger W, Kalinowski J, Thierbach G, Pühler A. Small mobilizable multi-purpose cloning vectors derived from the *Escherichia coli* plasmids pK18 and pK19: selection of defined deletions in the chromosome of *Corynebacterium glutamicum*. Gene. 1994;145: 69–73.

10. Monds RD, Newell PD, Gross RH, O’Toole GA. Phosphate-dependent modulation of c-di-GMP levels regulates *Pseudomonas fluorescens* Pf0-1 biofilm formation by controlling secretion of the adhesin LapA. Mol Microbiol. 2007;63: 656–79. doi:10.1111/j.1365-2958.2006.05539.x

11. Newell PD, Monds RD, O’Toole GA. LapD is a bis-(3’,5')-cyclic dimeric GMP-binding protein that regulates surface attachment by *Pseudomonas fluorescens* Pf0-1. Proc Natl Acad Sci U S A. 2009;106: 3461–6. doi:10.1073/pnas.0808933106

12. Hanahan D. Studies on transformation of *Escherichia coli* with plasmids. J Mol Biol. 1983;166: 557–80.

13. López CM, Rholl DA, Trunck LA, Schweizer HP. Versatile dual-technology system for markerless allele replacement in *Burkholderia pseudomallei*. Appl Environ Microbiol. 2009;75: 6496–503. doi:10.1128/AEM.01669-09

1. Kovach ME, Elzer PH, Hill DS, Robertson GT, Farris MA, Roop RM, et al. Four new derivatives of the broad-host-range cloning vector pBBR1MCS, carrying different antibiotic-resistance cassettes. Gene. 1995;166: 175–6.
2. Simon R, Priefer U, Pühler A. A broad host range mobilization system for in vivo genetic engineering: transposon mutagenesis in gram negative bacteria. Nat. Biotechnol. 1983;1:784–91.
