# Supplementary material for: Homologs of the LapD-LapG c-di-GMP Effector System Control Biofilm Formation by Bordetella bronchiseptica
Source: PLoS One. 2016 Jul 5;11(7):e0158752. doi: 10.1371/journal.pone.0158752 (PMC4933386; doi:10.1371/journal.pone.0158752)
Supplement: S2 Table — (DOCX) [file pone.0158752.s005.docx]

**Table S2. Oligonucleotide primers used in this study.**

| **N°** | **Sequence 5´- 3´** | **Relevant**  **Information** |
| --- | --- | --- |
| 1 | TACCCATACGATGTTCCAGATTACGCTTAGCCCGGGATGCCATTGTCCACCCGTCCCCGTAA | Upstream flank for lapA KO Fwd |
| 2 | AACAGCTATGACCATGATTACGAATTCGAGCTCGGTACCCACCAGCGCGAAGGCGAACAA^a^ | Upstream flank for lapA KO Rev |
| 3 | CCCGGGCTAAGCGTAATCTGGAACATCGTATGGGTAGCAGGCGTACGGGTGGAAAT | Downstream flank for lapA KO Fwd |
| 4 | CCAAGCTTGCATGCCTGCAGGTCGACTCTAGAGGATCCCCCGAGGTTGAGTATGCACGCCACT | Downstream flank for lapA KO Rev |
| 5 | TACCCGTTTTTTTGGGCTAGCGAATTCGAGCTCGGTACCCATGGCCAATTCTTCCCCC | Mini-LapA N-terminus fwd |
| 6 | GCCAAGCTTGCATGCCTGCAGGTCGACTCTAGAGGATCCCCTTAGCGTAATCTGGAACATCGTATGGGTAGCCATTGGCCGTG | Mini-LapA N-terminus rev |
| 7 | CTGTTTTATCAGACCGCTTCTGCGTTCTGATGGAACCAGGCCGGCACGCTG | Upstream flank for lapG KO Fwd |
| 8 | CCTGCTGAGCCTGGCTTGCGTATAGGATCCTGCGCATGGAGCGCGAG | Upstream flank for lapG KO Rev |
| 9 | CTCGCGCTCCATGCGCAGGATCCTATACGCAAGCCAGGCTCAGCAGG | Downstream flank for lapG KO Fwd |
| 10 | CAATTTCACACAGGAAACAGCTATGTATAGAATTCGCTGCTGCAGGGCGAGAACAG | Downstream flank for lapG KO Rev |
| 11 | ATACCCGTTTTTTTGGGCTAGCGAATTCGAGCTCGGTACCCATGCCATTGTCCACCCGTCCCCGTAA | lapG fwd |
| 12 | GCCAAGCTTGCATGCCTGCAGGTCGACTCTAGAGGATCCCCCAGTCGACGAAGTATGGACATAGG | lapG rev |
| 13 | ATACCCGTTTTTTTGGGCTAGCGAATTCGAGCTCGGTACCCTTTCTCGCTGATCTCGCTGG | lapD fwd |
| 14 | GCCAAGCTTGCATGCCTGCAGGTCGACTCTAGAGGATCCCCTGTTCTCCAGCCACATTCCC | lapD rev |
| 15 | GCTGCTGCAGGGCGAGAACAG | Intergenic LapA/G Fw |
| 16 | CGCAAGCCAGGCTCAGCAGG | Intergenic LapA/G Rev |
| 19 | AAGACCACGCTCACGCTGCAGGTCA | recA Fw |
| 20 | ATCAGCAGGTCGGTCAGGTTGACGC | recA Rev |

^a^Underlined sequences denote non-annealing bases employed for recombination in yeast
