# Supplementary material for: Homologs of the LapD-LapG c-di-GMP Effector System Control Biofilm Formation by Bordetella bronchiseptica
Source: PLoS One. 2016 Jul 5;11(7):e0158752. doi: 10.1371/journal.pone.0158752 (PMC4933386; doi:10.1371/journal.pone.0158752)
Supplement: S3 Table — (PDF) [file pone.0158752.s006.pdf]

Host	*B. bronchiseptica* strain	Number of repeats (VCBS + CADG)
**Cat**	MBORD635	unknown
**Cat**	MBORD782	unknown
**Dog**	MBORD785	unknown
**Dog**	MBORD839	unknown
**Dog**	MBORD591	unknown
**Dog**	MBORD595	unknown
**Guinea pig**	MBORD665	unknown
**Guinea pig**	MBORD668	unknown
**Guinea pig**	MBORD670	2
**Guinea pig**	MBORD678	unknown
**Guinea pig**	MBORD762	2
**Horse**	7E71	5
**Horse**	MBORD624	3
**Horse**	MBORD632	2
**Horse**	MBORD731	4
**Human**	1289	unknown
**Human**	Bbr77	5
**Human**	MOI 149	11
**Human**	253	13
**Human**	00-P-2730	unknown

Host	*B. bronchiseptica* strain	Number of repeats (VCBS + CADG)
Human	F4563	3
Human	GA96-01	unknown
Human	MBORD675	3
Human	MO211	unknown
Human	MO275	unknown
Human	SBL-F6116	unknown
Koala	MBORD681	unknown
Koala	MBORD698	3
Pig	CARE970018BB	4
Pig	MBORD849	3
Rabbit	RB50	15
Rabbit	3E44	6
Rabbit	A1-7	unknown
Rabbit	B18-5 (C3)	6
Rabbit	B20-10725633	7
Rabbit	RB630	unknown
Sea otter	SO10328	3
Seal	M435/02/3	2
Seal	M85/00/2	unknown
Turkey	CA90 BB02	unknown

Host	*B. bronchiseptica* strain	Number of repeats (VCBS + CADG)
**Human**	00-P-2796	2
**Human**	345	3
**Human**	D756	unknown
**Human**	D989	5
**Human**	D993	unknown
**Human**	E010	2
**Human**	E012	2
**Human**	E013	unknown
**Human**	E014	3

Host	*B. bronchiseptica* strain	Number of repeats (VCBS + CADG)
Turkey	CA90 BB1334	unknown
Turkey	F-1	3
Turkey	F2	3
Turkey	MBORD707	5
Turkey	MBORD901	3
Turkey	OSU054	unknown
Turkey	OSU095	3
Turkey	OSU553	unknown
unknown	980	unknown
