# Supplementary material for: Homologs of the LapD-LapG c-di-GMP Effector System Control Biofilm Formation by Bordetella bronchiseptica
Source: PLoS One. 2016 Jul 5;11(7):e0158752. doi: 10.1371/journal.pone.0158752 (PMC4933386; doi:10.1371/journal.pone.0158752)
Supplement: S1 Text — (DOCX) [file pone.0158752.s007.docx]

**Detailed Materials and Methods**

Yeast cloning for plasmid construction:

All oligonucleotide primers used in the study are listed in Table S2. Unless otherwise noted, cloning was performed by in vivo recombination in yeast, as described [Shanks *et al*., 2006]. Briefly, vectors bearing the 2μ replication origin and URA3 gene were linearized by restriction enzyme digestion. One or more inserts were generated by PCR using primers with 30 or more non-annealing bases to facilitate homologous recombination with adjacent fragments and/or the vector. Then, PCR fragments and linearized vector were introduced into yeast by transformation and recombinant vector bearing insert(s) of interest was selected for on uracil minus medium. All PCR was performed using Phusion polymerase (Finzymes).

Plasmid and strain construction

Plasmids based on the pK18mob-SacB allelic replacement vector, were used to generate Δ*lapG and ΔlapA* knockout strains in WT backgrounds. The same basic technique was employed in all cases. Two stretches of homologous DNA flanking the genomic region to be deleted were amplified by PCR (700-1000bp each), utilizing primers with 30 or more extra bases to facilitate recombination with adjacent fragments in cloning. Yeast cloning techniques was employed to introduce both fragments in pMQ30 plasmid. These plasmids were routinely used for allelic replacement in *P. fluorescens*. However SacB-positive selection in *B. bronchiseptica* was not successful for unknown reasons in our studies. Cloned fragments were transferred by classical molecular techniques to pK18mob-SacB to make allelic replacement experiments.

Allelic replacement

Briefly, constructs were introduced into *B. bronchiseptica* by conjugation with *E. coli*, and transconjugates selected for on BGA 80 mg ml^-1^ Kanamycin and 200 mg ml^-1^ Streptomycin. After verifying single-crossover insertion of constructs in the chromosome by PCR, strains were cultured O/N without antibiotic, then plated on Steiner Scholet media with 15.0 % (wt/vol) sucrose to select for the second crossover event. Mutants were verified by PCR and sequencing of purified genomic DNA to ensure proper construction.

Construction of expression plasmids

For expression in *E. coli* or *P. fluorescens*, constructs were built in pMQ72 downstream of the P_BAD_ promoter to yield pMQ72*lapG*_Bb_. Native sequences were amplified by PCR from genomic DNA using primers with additional non-annealing bases to facilitate recombination with the vector during yeast cloning. For expression in *B. bronchiseptica* background, the *lapG* gene was subcloned from pMQ72*lapG*Bb by classical techniques to broad host range plasmid pBB1MCS-5 with a constitutive promoter *npt*II (Sisti *et al.*, 2013). All variants were confirmed by DNA sequencing.

Construction of GFP tagged strains.

Insertion in *Tn*7 genomic site of *gfp* gene was employed to tagged *B. bronchiseptica* strains. pGFLIP plasmid was introduced by conjugation and conjugants were selected using kanamicin (80 mg ml^-1^).

**Supporting Information References**

Shanks RM, Caiazza NC, Hinsa SM, Toutain CM, O'Toole GA (2006) *Saccharomyces cerevisia*e-based molecular tool kit for manipulation of genes from gram-negative bacteria. Appl Environ Microbiol 72: 5027-5036.
